# Ambrisentan Exhibits Hepatoprotective Effects Against NASH-Associated Hepatic Injury in Dexamethasone-Treated Rats Through Regulation of Inflammation, Ferroptosis and Autophagy

**DOI:** 10.3390/ph19050798

**Published:** 2026-05-20

**Authors:** Naif S. Alharbi, Manar A. Nader, Marwa S. Serrya, Marwa E. Abdelmageed

**Affiliations:** 1 Department of Pharmacology and Toxicology, Faculty of Pharmacy, Mansoura University, ElGomhoria Street, Mansoura 35516, Egypt; 2 Assistant Deputy Ministry for Human Resources Services, Ministry of Health, Madinah, Saudi Arabia; 3 Department of Pharmacology and Toxicology, Faculty of Health Science Technology, Mansoura National University, Gamasa 7731168, Egypt

**Keywords:** autophagy, dexamethasone, NASH, oxidative stress, ferroptosis

## Abstract

**Background/Objectives:** Non-alcoholic steatohepatitis (NASH) represents a worldwide health challenge with limited currently available effective treatment. The present analysis was designed to examine possible therapeutic advances of Ambrisentan (AMB) targeting multiple features of hepatic damage in dexamethasone (DEXA)-provoked nonalcoholic steatohepatitis (NASH) in rats. **Methods:** Rats were randomly divided into four groups: a control group; a DEXA group; and two AMB-treated groups that received AMB (5 or 10 mg/kg/day orally for a week) before and concomitantly with DEXA (8 mg/kg/day, i.p.) for 6 days. After completion of the experiment, serum markers of liver function and lipid profile were assessed, and hepatic histopathological alterations were examined. **Results:** AMB (mainly at 10 mg/kg/day) markedly ameliorated liver-function parameters, the lipid profile, and hepatic histopathological characteristics in DEXA-treated rats. MDA was reduced, whereas GSH, GPX4 and Nrf2 were heightened, indicating elevated oxidative damage. Moreover, AMB efficiently reinstated iron homeostasis and aggravated iron overload by altering serum iron, hepatic ferritin, transferrin and hepcidin. AMB decreased serum calcium and hepatic calcineurin A levels, followed by a reduction in hepatic autophagy biomarker Beclin-1. AMB downregulated pro-inflammatory biomarkers NF-κB, IL-6 and TGF-β1. Moreover, it notably repressed the hepatic gene expression of ferritinophagy biomarker NCOA4, with elevated FTH1 hepatic gene expression. Moreover, AMB ameliorated DEXA-induced changes in endothelial and vascular function by increasing hepatic PGI2 and cGMP and lowering ET-1 and iNOS. **Conclusions:** AMB improved DEXA-induced NASH, primarily through its action on endothelin pathways, with associated reductions in inflammation and the downstream processes of ferroptosis, ferritinophagy, lipophagy, and autophagy.

## 1. Introduction

An advanced and rigorous stage of non-alcoholic fatty liver disease (NAFLD) is non-alcoholic steatohepatitis (NASH). Some of the symptoms include hepatic steatosis; inflammation; and more advanced stages of fibrosis, including stage 1 to stage 4, which may cause cirrhosis, liver failure, or hepatocellular carcinoma. Comorbidities linked with NASH include obesity, metabolic syndrome, and type 2 diabetes mellitus (T2DM), making it a worldwide health concern [1,2,3].

The “multiple hit” theory clarifies NASH pathogenesis well, integrating genetic, metabolic, and environmental aspects, incorporating lipotoxicity, oxidative stress, gut dysbiosis, and immune stimulation, which jointly drive inflammation and fibrosis [4,5,6,7,8,9,10].

Emerging evidence suggests that NASH is more than just a metabolic disorder; it is a complex, multifactorial disease involving many interacting cellular and molecular pathways. Concomitant endothelial dysfunction—in particular, dysfunction of liver sinusoidal endothelial cells (LSECs)—further promotes disease progression. In a healthy state, LSECs maintain hepatic homeostasis by controlling blood flow and immune cell trafficking and inhibiting hepatic stellate cell (HSC) activation. In NASH, LSECs become vascularized, lose fenestrae, and show reduced nitric oxide availability and enhanced expression of adhesion molecules, thereby promoting leukocyte recruitment and inflammatory signaling [11]. Oxidative stress is considered one of the driving forces in NASH progression and is associated with neutrophil infiltration and liver injury in clinical samples. Hepatic inflammation and injury induced in mice by a methionine- and choline-deficient diet were associated with the formation of IgG against malondialdehyde (MDA) and 4-hydroxynonenal (4-HNE) adducts, alongside infiltration of CD4^+^ and CD8^+^ T cells. These observations imply that oxidative stress promotes NASH through hepatocyte injury, as well as through the activation of adaptive immune responses [12]. These vascular modifications highlight the crucial role of hepatic endothelial dysfunction as a dynamic driver of NASH deterioration rather than a secondary consequence.

The current standard therapies for NASH are focused on lifestyle changes, including diet restriction and physical activities. However, the reliance on lifestyle modification alone presents significant challenges in real-world settings, as sustained adherence to lifestyle modification is often difficult to maintain, limiting long-term effectiveness. Common current therapies also include some off-label pharmacological therapies targeting the metabolic aspects of NASH, including pioglitazone and vitamin E, which are not very effective and focus more on the metabolic factors in NASH but not vascular pathology, which is the active driver of NASH disease progression [1]. Additionally, these treatments are not approved by the FDA for NASH, and their long-term use can be linked with side effects such as weight gain with pioglitazone or cardiovascular risk with vitamin E, as reported by Sanyal and his team [13]. Recently, GLP-1 receptor agonists—particularly, semaglutide—have attracted considerable attention. In terms of the histologic resolution of NASH and related metabolic benefits, this class has shown strong efficacy, with yet unmet needs [8,14].

Ambrisentan (AMB) is an endothelin antagonist with vasoprotective and anti-fibrotic properties. Endothelin-1 (ET-1) is a potent peptide vasoconstrictor that supports the proliferation of smooth muscle cells. It was found that ET-1 is expressed in the liver, suggesting its participation in the activation of hepatic stellate cells, vascular resistance, and inflammation, key processes in the progression of NASH. AMB may decrease portal hypertension and enhance liver function. It has been found that AMB may lower hepatic vascular resistance and inflammation and, therefore, may act as a new treatment option for conditions like NASH [15,16]. Moreover, the targeting of endothelin signaling seems favorable, since it can help improve metabolic aspects of NASH [17]. Despite these developments, the role of the endothelin signaling pathway in relating vascular dysfunction to metabolic and inflammatory pathways in NASH is still unsatisfactorily investigated, signifying a promising yet under-investigated therapeutic target.

Dexamethasone (DEXA) is a derivative of glucocorticoids used to treat different inflammatory disorders [18,19]. Different experimental studies have specified how treatment with DEXA leads to the development of NASH-like phenotypes [1,20]. The assortment of DEXA-provoked NASH is specifically significant from a translational perspective, as glucocorticoids are extensively used in clinical practice for the management of inflammatory and autoimmune diseases. Sustained or high-dose use of DEXA is known to induce metabolic disturbances, insulin resistance, and hepatic steatosis, strictly imitating the main characteristics of NASH, thereby affording a clinically applicable experimental model.

Therefore, the purpose of this research is the assessment of the effectiveness of AMB in counteracting the effects of DEX-induced NASH, with a precise emphasis on its ability to moderate the endothelin signaling pathway and its downstream effects on hepatic inflammation, metabolic dysregulation, and fibrosis.

## 2. Results

### 2.1. Effect of AMB (5 and 10 mg/kg) on DEXA-Induced Changes in Liver Function

**Figure 1:**
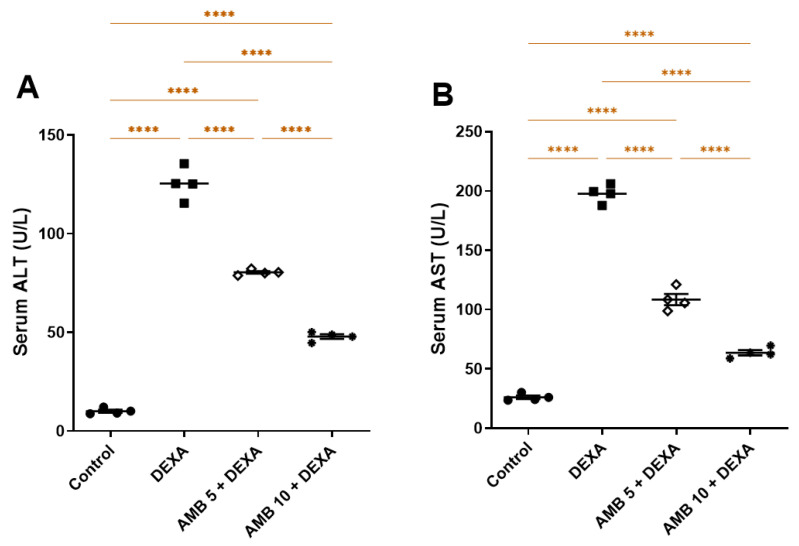
Impact of AMB on NASH-provoked liver-function changes in rats. (**A**) Serum ALT; (**B**) serum AST. Means ± SEM (n = 4) were utilized to express data using one-way ANOVA, followed by Tukey’s post hoc multiple-comparison test (**** *p* < 0.0001). DEXA: dexamethasone; AMB: ambrisentan; ALT: alanine aminotransferase; AST: aspartate aminotransferase.

Serum ALT and AST levels were markedly elevated in the DEXA-treated group compared to the controls. Both AMB doses significantly reduced serum ALT and AST levels compared to the DEXA group in a dose-dependent manner (Figure 1A,B).

### 2.2. Effect of AMB (5 and 10 mg/kg) on DEXA-Induced Histopathological Changes

**Figure 2:**
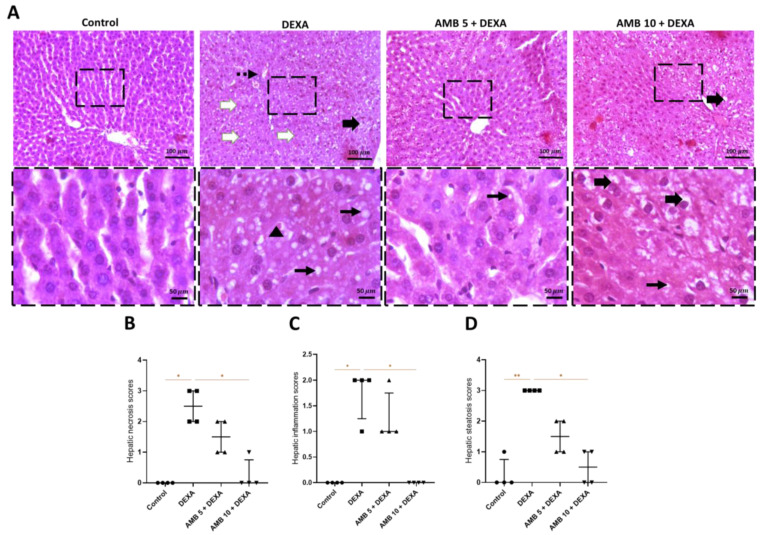
Impact of AMB on NASH-provoked pathological alterations in rat livers. (**A**) Illustrative liver-sector photos stained with H&E stain; (**B**) necrosis score; (**C**) inflammation score; (**D**) steatosis score. Thin arrow = steatosis; dashed arrow = inflammation; thick white arrows = necrosis; thick black arrows = hydropic degeneration; arrowheads = apoptotic bodies. Image magnification = 100×, 400×. Medians and interquartile ranges (n = 4) were utilized to express data using the Kruskal–Wallis test followed by Dunn’s multiple-comparison test (* *p* < 0.05, ** *p* < 0.01). DEXA: dexamethasone; AMB: ambrisentan.

As shown in Figure 2A, H&E-stained liver sections from the control group exhibited normal hepatic architecture. In contrast, the diseased group demonstrated marked necrosteatohepatitis, with diffuse cytoplasmic microvesicular steatosis; hydropic degeneration; many eosinophilic shrunken apoptotic bodies with scattered hepatocellular necrosis; and low to mild perivascular inflammatory infiltrates composed of low numbers of lymphocytes, macrophages, and occasional neutrophils admixed with few fibroblasts. Sectors from the AMB (5 mg/kg) group showed minimal steatosis characterized by few intracytoplasmic microvesicles. The AMB (10 mg/kg) group showed coalescing hepatocellular degeneration with pale, swollen, vacuolated hepatocytes admixed with a limited number of intracytoplasmic vacuoles.

### 2.3. Effect of AMB (5 and 10 mg/kg) on DEXA-Induced Hepatic Ultrastructural Changes Determined Using Electron Microscopy

**Figure 3:**
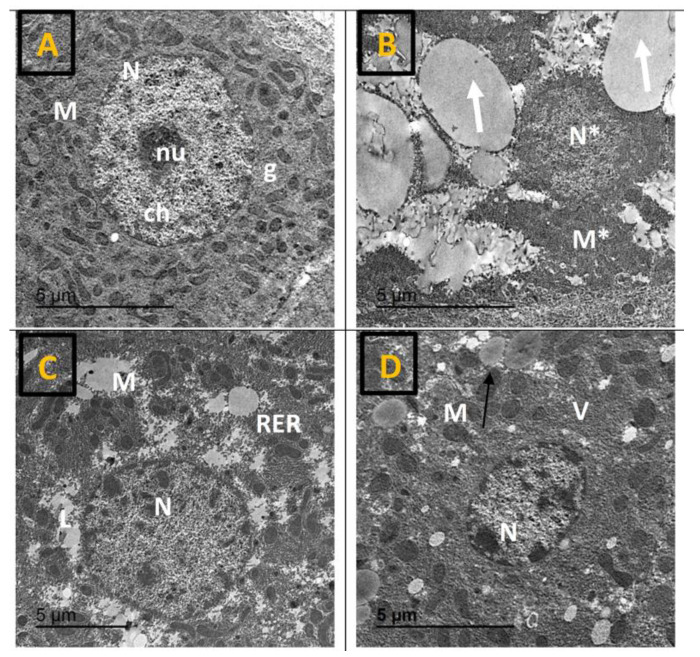
Impact of AMB on NASH-provoked ultrastructural liver alteration in rats. Transmission electron micrographs of hepatocytes. (**A**) Control group; (**B**) DEXA group; (**C**) AMB 5 + DEXA; (**D**) AMB 10 + DEXA. L: fine lipid droplets; N: normal nucleus; N*: irregular/pyknotic nucleus; nu: nucleolus; M: normal mitochondria; M*: degenerated mitochondria; RER: rough endoplasmic reticulum; V: vacuoles; ch: chromatin clusters; thick white arrow: large lipid droplets; thin black arrow: lysosomes. Magnification: 20,000×.

TEM graphs of hepatocytes from the control group revealed normal cellular architecture, showing hepatocytes with a single round, euchromatic nucleus containing scattered chromatin clusters and a prominent nucleolus. The cytoplasm displayed abundant mitochondria with a spherical or rod-shaped morphology, well-organized rough endoplasmic reticulum (rER), and large glycogen aggregates interspersed among intact organelles (Figure 3A). In contrast, hepatocytes from the DEXA group demonstrated profound ultrastructural deterioration, including loss of normal cellular architecture, irregular and pyknotic nuclei, numerous lipid droplets, markedly degenerated and electron-dense mitochondria, and dilated rER. Increased lysosomal content was also evident, indicating active cellular injury and degeneration (Figure 3B). Treatment with AMB (5 mg/kg) resulted in partial improvement of DEXA-induced changes, with hepatocytes showing less pronounced nuclear irregularities, fewer vacuoles, and mitochondria of mostly normal appearance (Figure 3C). AMB (10 mg/kg) exhibited near-complete restoration of hepatocyte ultrastructure, characterized by a nearly normal nucleus, a cytoplasm containing only a few small vacuoles, and mitochondria showing minimal swelling (Figure 3D).

### 2.4. Effect of AMB (5 and 10 mg/kg) on DEXA-Induced Iron Homeostasis Imbalance

**Figure 4:**
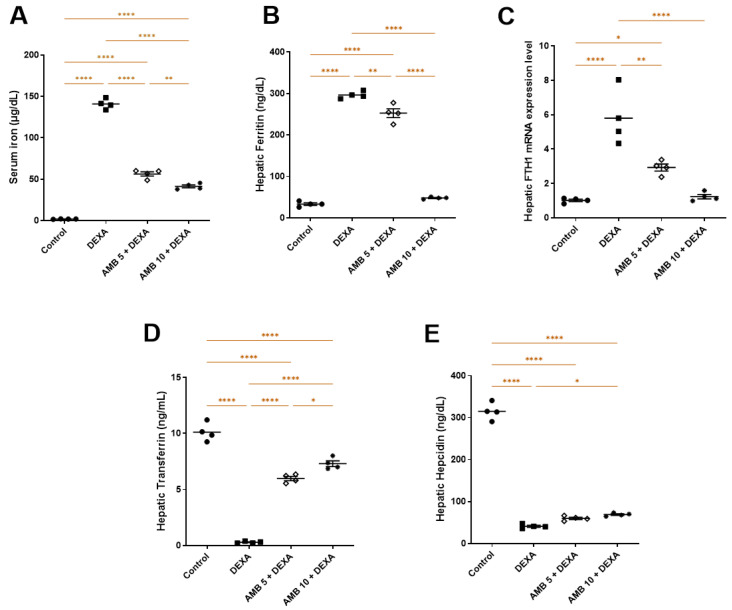
Impact of AMB on NASH iron homeostasis imbalance. (**A**) Serum iron; (**B**) hepatic ferritin; (**C**) FTH1 mRNA expression; (**D**) transferrin; (**E**) hepcidin. Means ± SEM (n = 4) were utilized to express data using one-way ANOVA, followed by Tukey’s post hoc multiple-comparison test (* *p* < 0.05, ** *p* < 0.01, **** *p* < 0.0001). DEXA: dexamethasone; AMB: Ambrisentan; FTH1: ferritin heavy chain.

Rats treated with DEXA showed a significant increase in serum iron compared with the control group, whereas AMB-treated groups exhibited marked reductions in serum iron relative to DEXA (Figure 4A). Regarding hepatic iron homeostasis markers, DEXA administration disrupted ferritin, transferrin, hepcidin, and FTH1 levels, as evidenced by elevated hepatic ferritin and FTH1 gene expression alongside significantly reduced transferrin and hepcidin compared with controls. In contrast, both doses of AMB meaningfully lessened hepatic ferritin and FTH1 expression while increasing transferrin and hepcidin levels relative to the DEXA group (Figure 4B–E). Overall, AMB dose-dependently counteracted DEXA-induced disturbances in hepatic iron homeostasis, highlighting its protective effect.

### 2.5. Effect of AMB (5 and 10 mg/kg) on DEXA-Induced Changes in Hepatic Oxidative Stress

**Figure 5:**
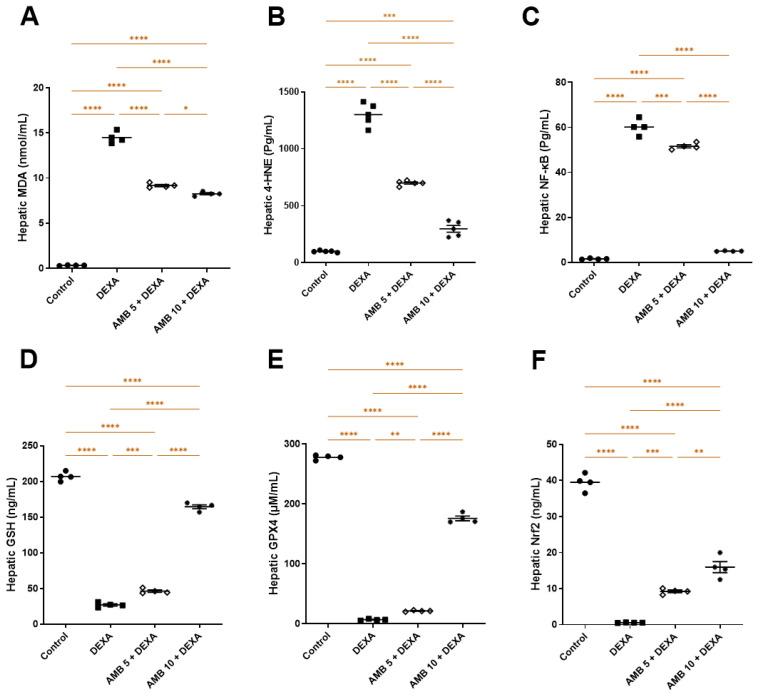
Impact of AMB on NASH-induced oxidative imbalance in rat liver. (**A**) Hepatic MDA; (**B**) hepatic 4-HNE; (**C**) Hepatic NF-κB; (**D**) hepatic GSH; (**E**) hepatic GPX4; (**F**) hepatic Nrf2. Means ± SEM (n = 4) were utilized to express data using one-way ANOVA, followed by Tukey’s post hoc multiple-comparison test (* *p* < 0.05, ** *p* < 0.01, *** *p* < 0.001, **** *p* < 0.0001). DEXA: dexamethasone; AMB: ambrisentan; NF-κB: nuclear factor kappa B; MDA: malondialdehyde; GSH: glutathione; GPX4: glutathione peroxidase-4; Nrf2: nuclear factor erythroid 2–related factor 2; 4-HNE: 4-hydroxynonena.

DEXA-treated rats exhibited pronounced hepatic oxidative stress characterized by increased lipid peroxidation and depletion of antioxidant defenses. Specifically, DEXA significantly elevated hepatic MDA, 4-HNE, and NF-κB levels while markedly reducing Nrf2, GSH, and GPX4 compared with controls. In contrast, AMB administration significantly restored the oxidant–antioxidant balance, with the 10 mg/kg dose showing a greater effect than 5 mg/kg when compared with the DEXA group (Figure 5A–E).

### 2.6. Effect of AMB (5 and 10 mg/kg) on DEXA-Induced Changes in the Ferritinophagy

**Figure 6:**
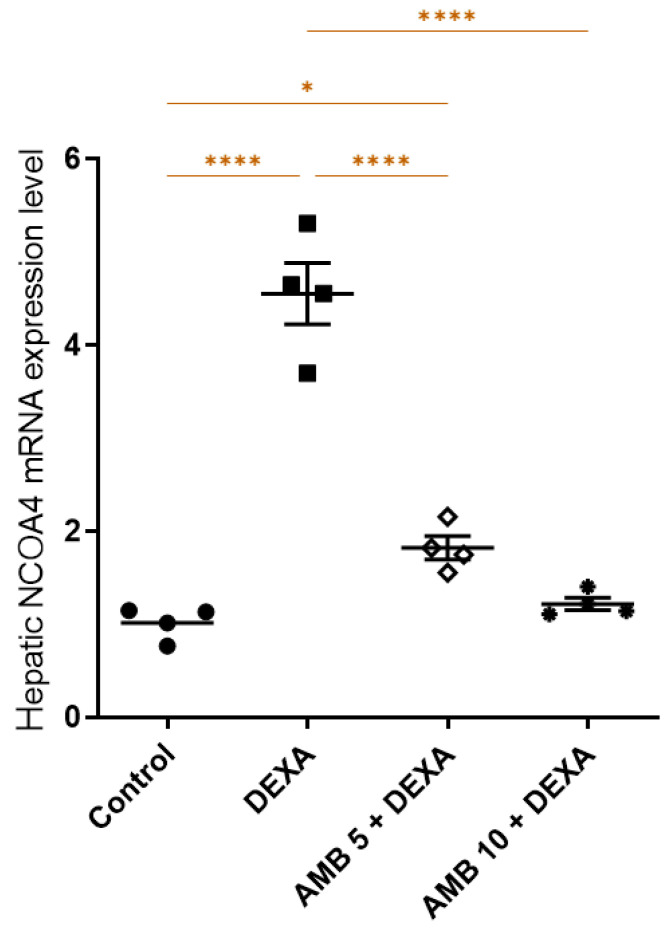
Impact of AMB on NASH-induced changes in hepatic NCOA4 levels in rats. Means ± SEM (n = 4) were utilized to express data using one-way ANOVA, followed by Tukey’s post hoc multiple-comparison test (* *p* < 0.05, **** *p* < 0.0001). DEXA: dexamethasone; AMB: ambrisentan; NCOA4: nuclear receptor coactivator-4.

Figure 6 demonstrates that DEXA significantly upregulated hepatic NCOA4 gene expression compared to controls, indicating activation of the ferritinophagy biomarker. AMB administration at both doses significantly reduced hepatic NCOA4 gene expression compared with the DEXA-treated groups.

### 2.7. Effect of AMB (5 and 10 mg/kg) on DEXA-Induced Autophagy

**Figure 7:**
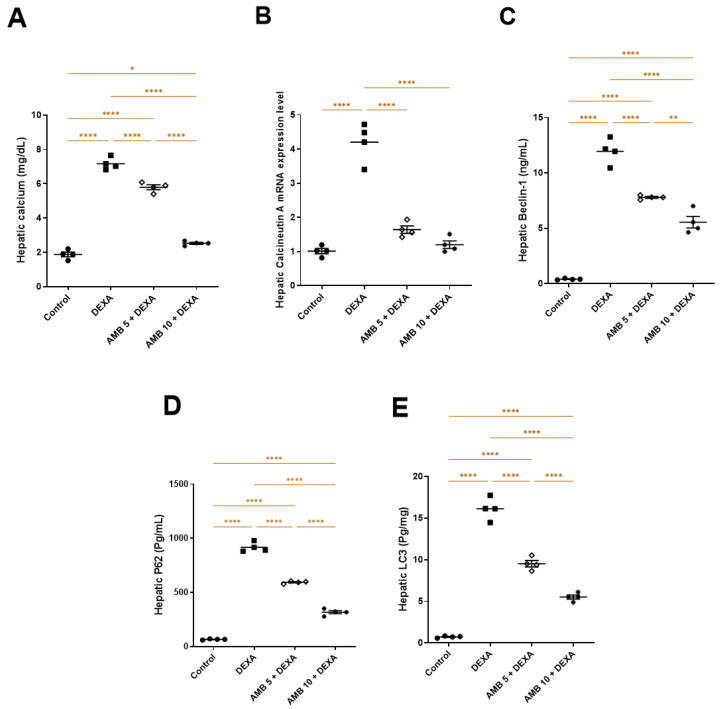
Impact of AMB on NASH-induced autophagy in rats. (**A**) Serum calcium; (**B**) hepatic calcineurin; (**C**) hepatic beclin-1; (**D**) hepatic p62; (**E**) hepatic LC3-II. Means ± SEM (n = 4) were utilized to express data using one-way ANOVA, followed by Tukey’s post hoc multiple-comparison test (* *p* < 0.05, ** *p* < 0.01, **** *p* < 0.0001). DEXA: dexamethasone; AMB: ambrisentan.

Rats administered DEXA showed significant increases in serum calcium, hepatic calcineurin A mRNA expression, hepatic beclin-1, p62, and LC3-II levels compared with the control group. AMB treatment significantly modulated these autophagy-related markers in a dose-dependent manner, as evidenced by marked reductions in serum calcium; calcineurin A expression; and beclin-1, p62, and LC3-II levels relative to the DEXA group (Figure 7A–E).

### 2.8. Effect of AMB (5 and 10 mg/kg) on DEXA-Induced Lipophagy

**Figure 8:**
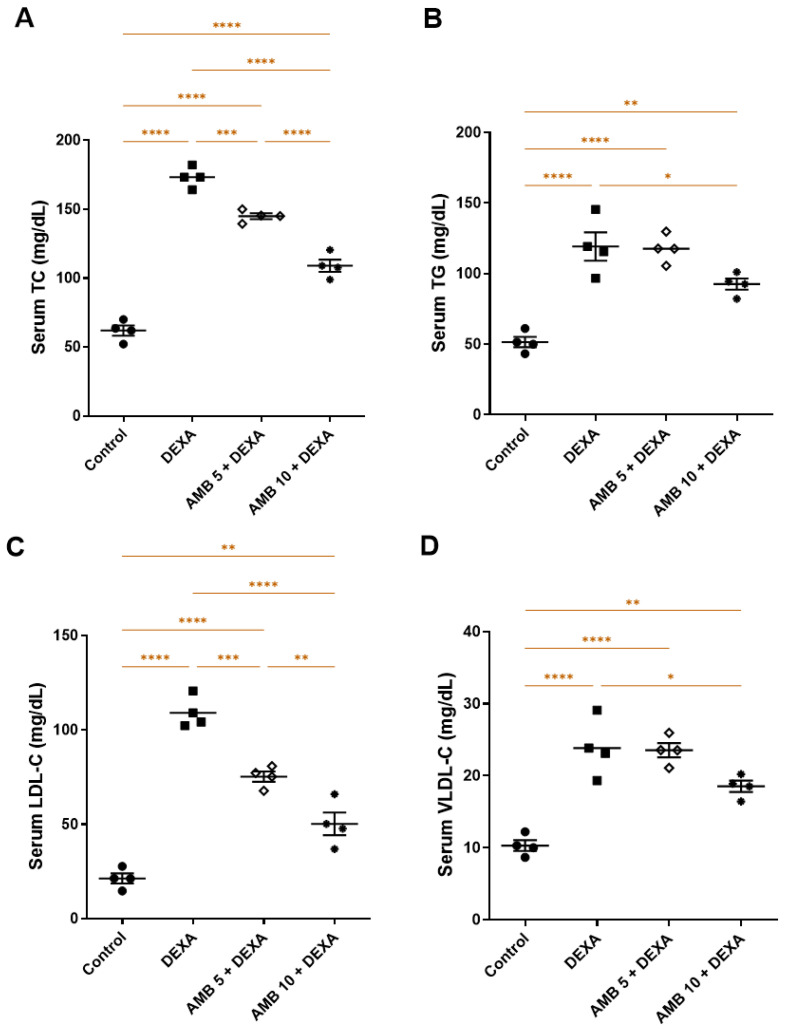
Impact of AMB on NASH-induced lipophagy in rats. (**A**) Serum TC; (**B**) serum TG; (**C**) serum LDL-C; (**D**) serum VLDL-C. Means ± SEM (n = 4) were utilized to express data using one-way ANOVA, followed by Tukey’s post hoc multiple-comparison test (* *p* < 0.05, ** *p* < 0.01, *** *p* < 0.001, **** *p* < 0.0001). DEXA: dexamethasone; AMB: ambrisentan; TC: total cholesterol; TG: triglycerides; LDL-C: low-density lipoprotein cholesterol; VLDL-C: very low-density lipoprotein cholesterol.

DEXA administration significantly worsened the lipid profile, as evidenced by marked elevations in serum TG, TC, LDL-C, and VLDL-C compared with the control group. In contrast, both doses of AMB significantly reduced these lipid parameters relative to the DEXA group. Notably, the higher dose showed a more pronounced effect in lowering TC and LDL-C levels (Figure 8A–D).

### 2.9. Effect of AMB (5 and 10 mg/kg) on DEXA-Induced Inflammation and Fibrosis

**Figure 9:**
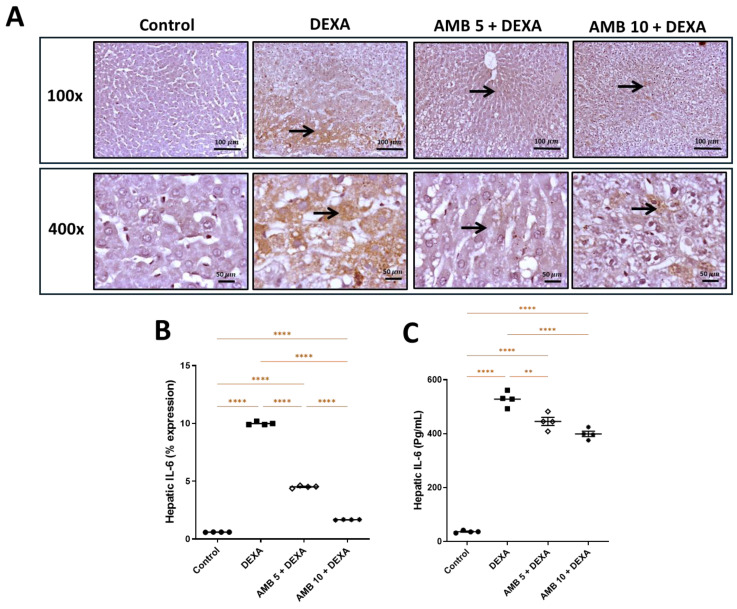
Impact of AMB on NASH-induced changes in IL-6 in rat liver. (**A**) IHC photos for IL-6 expression level; (**B**) hepatic IL-6% expression; (**C**) hepatic IL-6 level. Arrow indicates positive stained hepatocytes. Means ± SEM (n = 4) were utilized to express data using one-way ANOVA, followed by Tukey’s post hoc multiple-comparison test (** *p* < 0.01, **** *p* < 0.0001). DEXA: dexamethasone; AMB: ambrisentan; IL-6: interleukin-6.

**Figure 10:**
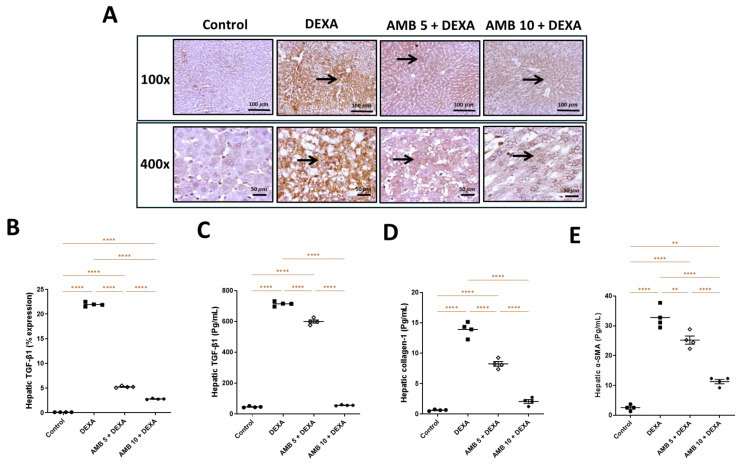
Impact of AMB on NASH-induced changes in TGF-β1, collagen-1 and α-SMA levels in rat liver. (**A**) IHC photos for hepatic TGF-β1 expression level; (**B**) hepatic TGF-β1% expression; (**C**) hepatic TGF-β1; (**D**) hepatic collagen-1; (**E**) hepatic α-SMA. Arrow indicates positively immuno stained hepatocytes. Means ± SEM (n = 4) were utilized to express data using one-way ANOVA, followed by Tukey’s post hoc multiple-comparison test (** *p* < 0.01, **** *p* < 0.0001). DEXA: dexamethasone; AMB: ambrisentan; TGF-β1: transforming growth factor beta 1; α-SMA: alpha smooth muscle actin.

DEXA significantly increased hepatic levels of IL-6, TGF-β1, collagen 1, and α-SMA compared with controls (Figure 9 and Figure 10). Conversely, AMB treatment reduced all these markers in a dose-dependent manner. IHC analysis revealed that IL-6 was minimally detected or not detected in controls but strongly expressed in DEXA-treated rats in both hepatocytes and inflammatory cells. AMB treatment significantly reduced IL-6 expression, with a greater reduction observed at the higher dose. Similarly, TGF-β1 expression was absent/weak in controls, strongly positive in the DEXA group, and progressively decreased in AMB-treated groups, with the higher dose showing the least staining. Quantitatively, DEXA markedly elevated TGF-β expression, while AMB significantly attenuated it (Figure 9 and Figure 10).

### 2.10. Effect of AMB (5 and 10 mg/kg) on DEXA-Induced Changes in Hepatic Endothelial and Vascular Function

**Figure 11:**
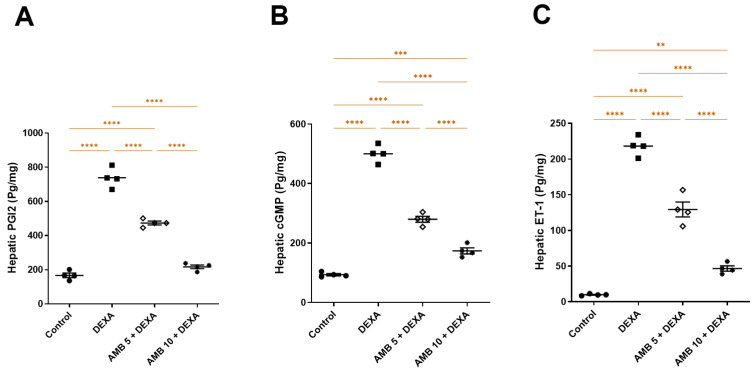
Impact of AMB on NASH-provoked endothelial and vascular alterations in rats. (**A**) Hepatic PGI2; (**B**) hepatic cGMP; (**C**) hepatic ET-1. Means ± SEM (n = 4) were utilized to express data using one-way ANOVA, followed by Tukey’s post hoc multiple-comparison test (** *p* < 0.01, *** *p* < 0.001, **** *p* < 0.0001). DEXA: dexamethasone; AMB: ambrisentan; PGI2: prostacyclin; cGMP: cyclic guanosine monophosphate; ET-1: endothelin-1.

DEXA administration caused significant disruptions in endothelial and vasoactive signaling, as evidenced by marked reductions in PGI2 and cGMP and elevated hepatic ET-1 levels compared with the control group. Both AMB doses significantly mitigated these alterations, with AMB 5 mg/kg producing clear ameliorations across all markers relative to the DEXA group. The higher AMB dose (10 mg/kg) exerted a more robust effect, restoring PGI2 levels to near-control values with further elevation in cGMP and lessening in ET-1 hepatic levels, although these remained slightly elevated compared with normal levels (Figure 11A–C).

**Figure 12:**
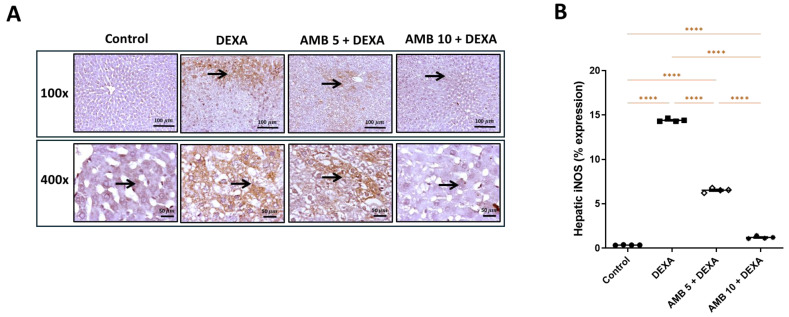
Impact of AMB on NASH-induced hepatic iNOS expression alteration in rats. (**A**) IHC photos for hepatic iNOS expression level; (**B**) iNOS % expression. Arrow indicates positively immunostained hepatocytes. Means ± SEM (n = 4) were utilized to express data using one-way ANOVA, followed by Tukey’s post hoc multiple-comparison test (**** *p* < 0.0001). DEXA: dexamethasone; AMB: ambrisentan; iNOS: inducible nitric oxide synthase.

The hepatic expression of iNOS indicated that the control group displayed negative immunostained hepatocytes. DEXA showed moderately to strongly immunopositive-stained hepatocytes; the AMB 5 + DEXA group showed moderately immunopositive-stained hepatocytes. The AMB 10 + DEXA group showed a few immunopositive-stained hepatocytes. (Figure 12A). Figure 12B reveals that the hepatic expression of iNOS was significantly elevated in the diseased rats compared to the controls, while treatment with AMB (5 or 10 mg/kg) significantly reduced hepatic iNOS expression compared to the diseased group.

Overall, the 10 mg/kg dose demonstrated superior efficacy in counteracting the DEXA-induced activation of endothelial mediators and stress-responsive transcriptional pathways.

## 3. Discussion

AMB, a selective endothelin-A receptor antagonist, exerted broad hepatoprotective effects by targeting ET-1-mediated vascular and inflammatory signaling. AMB markedly reduced ET-1 levels, leading to restoration of PGI_2_ and cGMP signaling and attenuation of endothelial stress, consistent with the established role of ET blockade in improving microvascular function [21,22,23]. This vascular protection was accompanied by significant suppression of IL-6, NF-κB, and TGF-β expression, indicating effective interruption of the ET-1-driven inflammatory–fibrotic axis that underlies NASH progression [24,25]. Given that ET-1 amplifies hepatic stellate-cell activation, cytokine release, and profibrotic signaling, AMB-mediated ET inhibition likely limits stellate-cell trans-differentiation and extracellular matrix deposition [26,27,28]. Collectively, these findings support a mechanistic framework in which AMB confers hepatoprotection by restoring endothelial homeostasis and suppressing downstream inflammatory and fibrogenic cascades central to NASH pathophysiology.

In the present work study, we effectively further recognized a NASH-like model illustrated by disturbed redox balance and iron homeostasis, laterally with enhanced inflammatory, fibrotic, and vascular changes [20]. DEXA exposure provoked a significant hepatic injury phenotype that was strongly linked with oxidative stress and redox imbalance. Increased hepatic MDA levels confirm elevated lipid peroxidation and membrane instability, revealing extreme ROS production. Simultaneous diminution of hepatic GSH, Nrf2, and GPX4 evokes a worsened antioxidant defense system and elevates vulnerability to oxidative damage, assuming the recognized effect of GPX4 in defending against lipid peroxidation [29]. Diminished GPX4 activity or depleted GSH levels render the cell more vulnerable to lipid peroxidation-induced ferroptosis [30,31]. These results may suggest a prospective influence of ferroptosis-associated mechanisms on liver injury.

Furthermore, DEXA processing was linked to obvious turbulence in hepatic iron homeostasis, as supported by diminished hepcidin and transferrin levels, as well as amplified serum iron and hepatic ferritin levels and upregulated FTH1 expression. These findings propose modified iron hemostasis and probable intracellular iron sequestration. The noted upregulation of NCOA4 expression may manifest stimulation of ferritinophagy-linked pathways, which could lead to the development of the labile iron pool. Excess ferrous iron encourages Fenton chemistry, causing enhanced ROS production and lipid peroxidation. In this setting, ferritinophagy may signify a possible mechanism relating iron dysregulation to oxidative stress and hepatocellular injury [28,32,33,34,35]. Glucocorticoids may suppress inflammatory signaling and hepcidin expression in specific contexts [36]. The current data support a dominant role for iron-dependent oxidative stress and ferroptosis in DEXA-induced hepatotoxicity, potentially reinforcing inflammatory cascades.

To further confirm the engrossment of ferroptosis and autophagy-linked mechanisms, additional markers were evaluated. The shown variations in LC3 and p62 levels postulate further insight into autophagic flux dynamics, as LC3-II buildup with p62 modulation indicates alterations in autophagosome construction and degradation [35,37]. The current results support the notion that autophagy was vigorously regulated in the present study.

In parallel, it is worth noting the rise of 4-HNE, a well-known end-product of lipid peroxidation [38], supporting the existence of oxidative membrane injury and reinforcing the involvement of lipid peroxidation-driven cell injury. Given that ferroptosis is related to iron-dependent lipid peroxidation, the mutual estimation of GPX4 diminution, augmented 4-HNE, and disturbed redox balance mutually advocates for the essential involvement of ferroptosis-related pathways.

Additionally, the integration of NCOA4 expression with autophagy markers (LC3 and p62) affords further support for the potential motivation of ferritinophagy, a selective form of autophagy that controls intracellular iron availability [39,40]. Nevertheless, it should be highlighted that current data are based on molecular and biochemical markers, and accordingly, additional work using more specific functional assays is needed to definitively ascertain mechanistic causality.

Hypertriglyceridemia and hypercholesterolemia developed because of DEXA administration. DEXA’s hyperlipidemic effects are probably caused by increased hepatic and intestinal VLDL production and decreased lipoprotein lipase activity. Additionally, DEXA raises free cholesterol levels, lowers lecithin cholesterol acyltransferase activity, and enhances hepatic lipogenesis and lipid deposition in the liver [41]. In the present study, DEXA induced profound dyslipidemia, as evidenced by increased TG, TC, LDL-C, and VLDL-C serum levels. Iron accumulation also increases the uptake of calcium ions [42]. The increase in calcium ions activates calcineurin, a protein phosphatase [43]. TFEB, an autophagy-promoting factor, is dephosphorylated in the cytosol, then translocated into the nucleus when calcineurin is activated [44]. By attaching to coordinated lysosomal expression and regulation (CLEAR) sequences, which are prevalent in many lysosome-related genes in hepatic cells, nuclear TFEB increases the expression of lysosome- and autophagy-related genes [44,45,46]. The transcriptional upregulation of calcineurin A and Beclin-1 (an autophagic protein) and heightened serum calcium levels signify aberrantly activated autophagy, along with an inability to recover lipid metabolism. Despite the apparent initiation of autophagy, defective lipophagic flux leads to the accumulation of lipid droplets, mitochondrial dysfunction, and hepatocellular lipotoxicity, thereby exacerbating steatosis and disease progression [47,48].

DEXA-induced inflammation was substantiated by marked upregulation of hepatic IL-6, NF-κB, and TGF-β, key mediators that collectively orchestrate immune activation, hepatic stellate-cell trans-differentiation, and fibrogenesis [49,50]. Persistent NF-κB activation promotes pro-inflammatory cytokine release, while IL-6 signaling exacerbates hepatocellular stress and metabolic dysregulation, thereby accelerating NASH progression [51,52]. Concurrent elevation of TGF-β further implicates activation of profibrotic pathways, reinforcing the transition from steatosis to fibrotic remodeling [53].

To further elucidate the existence of fibrosis, collagen-1 and α-SMA were assessed. The witnessed elevation in α-SMA postulates a powerful confirmation of hepatic stellate-cell stimulation, which represents a crucial event in the initiation and progression of liver fibrosis. Activated stellate cells are the initial source of extracellular matrix components, leading to fibrotic remodeling [54,55,56]. A concomitant increase in collagen levels further assists in enhanced extracellular matrix deposition, a hallmark of fibrogenesis [57,58]. Current data support the upregulation of TGF-β1, a central profibrotic cytokine known to drive stellate-cell activation and collagen synthesis.

Collectively, the mutual analysis of TGF-β1, α-SMA, and collagen postulates a more inclusive appraisal of fibrotic progression.

Moreover, the insulin resistance found in NAFLD may cause dysfunction in vascular endothelial function through multiple pathways, such as the disruption of nitric oxide production, resulting in reduced blood flow, which subsequently aggravates insulin resistance, thereby perpetuating a vicious cycle [59]. It has been demonstrated that oxidative stress disruption, which is frequently seen in NAFLD, affects metabolic inflammation by inducing vascular endothelial dysfunction, raising the risk of CVD in NAFLD patients [60].Vascular dysfunction caused by DEXA was evidenced by suppressed hepatic PGI_2_ and cGMP levels, together with increased hepatic ET-1 levels and hepatic iNOS expression, indicating profound endothelial imbalance and impaired sinusoidal perfusion [60,61]. Reduced PGI_2_–cGMP signaling compromises vasodilation and hepatocellular oxygen delivery, whereas ET-1 overexpression promotes vasoconstriction and intrahepatic vascular resistance [62]. Excessive iNOS-derived nitric oxide, particularly under oxidative conditions, favors peroxynitrite formation, amplifying nitrosative stress and endothelial injury [63].

AMB reinforced antioxidant defenses by restoring GSH, Nrf2, and GPX4 expression, thereby enhancing resistance to oxidative stress and ferroptosis [40,64]. Normalization of iron metabolism was evident from increased hepatic hepcidin and transferrin levels, with concomitant reductions in hepatic ferritin and hepatic FTH1 gene expression, reflecting improved iron handling and reduced intracellular iron sequestration [32,65]. Notably, suppression of NCOA4 suggests inhibition of ferritinophagy, thereby limiting ferritin degradation; labile iron release; and iron-mediated lipid peroxidation, a central driver of ferroptotic cell death [34]. Collectively, these effects highlight AMB as a potent modulator of redox balance, iron homeostasis, and ferroptosis-associated hepatocellular injury. Additionally, AMB corrected DEXA-induced autophagy and lipophagy dysregulation by normalizing hepatic calcineurin-A gene expression, as well as hepatic calcium and Beclin-1 levels, with improving lipid profiles. These effects indicate the restoration of effective autophagic flux and enhanced mitochondrial β-oxidation. Dose-dependent responses further support the therapeutic potential of endothelin receptor antagonism in metabolic liver disease.

Finally, in the current study, AMB was delivered at two doses (5 and 10 mg/kg) to assess dose-dependent effects. Though neither dose completely returned hepatic hepcidin, MDA, Nrf2, serum triglycerides, or VLDL-C levels to control values, both doses established variable levels of improvement compared with the DEXA group. The higher dose revealed a more noticeable modulatory effect than the lower dose, proposing a dose-dependent protective action. The incomplete restoration of parameters may be due to the rigorousness of DEXA-provoked metabolic and oxidative injury in the NASH rat model. These results revealed that the protective potential was vastly improved by AMB administration. Dose optimization and treatment duration may be required for maximal efficacy.

### Limitations of the Study

The current work has a few limitations that should be recognized. First, body weight was not systematically documented through an experimental study, which could have offered extra insights into the metabolic status and progression of the disease. Nonetheless, the induction of NASH-like characteristics was established via widespread histopathological, biochemical, and molecular evaluations. Second, though the DEXA rat model exhibited key characteristics of NASH, it signifies a pharmacological model that may not fully recapitulate all aspects of genetic or diet-induced disease models. Nevertheless, it is still highly appropriate to study glucocorticoid-persuaded metabolic alteration and related hepatic injury. Thirdly, the present study is based on indirect molecular markers of ferritinophagy activation and ferroptotic cell death, and the ultimate pivotal relationship between ferritinophagy activation and ferroptotic cell death cannot be convincingly proven. Also, while the DEXA-induced NASH model reproduces various significant properties of NASH, it may not fully capture the complexity of human NASH. Additionally, mechanistic and translational research is essential to further endorse these findings. Nevertheless, it should be noted that while measured fibrosis markers strongly indicate fibrogenesis, additional histopathological and molecular analyses could strengthen the confirmation of fibrosis severity in experimental NASH. Another limitation of the present study is that although AMB produced significant dose-dependent improvement in most assessed parameters, some biomarkers were not fully normalized to control levels, particularly those related to oxidative stress, lipid metabolism, and iron homeostasis. This may indicate that longer treatment durations, different dosing regimens, or combination strategies are required for complete restoration, which could be addressed in future investigations.

## 4. Materials and Methods

### 4.1. Animals and Ethical Approval

The current work was conducted using twenty-four adult male Wistar rats weighing between 200 and 250 g (obtained from VACSERA, Giza, Egypt). All animals were kept in a conventional laboratory environment, maintained at a controlled temperature of 22 ± 2 °C with a 12 h light/12 h dark cycle, and provided unrestricted access to food and water (*ad libitum*). The protocol was implemented in strict accordance with internationally recognized guidelines for the care and use of laboratory animals, as outlined by the Mansoura University Animal Care and Use Committee (MU-ACUC) under approval number (PHARM.PhD.23.12.32) and approval date (16th January 2024), in compliance with ethical and reporting standards of the ARRIVE guidelines.

### 4.2. Chemicals and Reagents

AMB was purchased from GILEAD Sciences, Inc. (Foster City, CA, USA). DEXA was secured from Amriya Pharmaceutical Industries (Alexandria, Egypt). The rest of the chemicals were of the finest grade.

### 4.3. Experimental Design

DEXA (8 mg/kg/day from day 8 to day 13, intraperitoneally (i.p.) administered) was utilized to provoke NASH. Additionally, AMB (5 mg/kg and 10 mg/kg) was prepared as a suspension in 0.5% carboxymethylcellulose (CMC) and taken orally. Rats were allocated into four groups (n = 4). The control group received CMC (2.5 mL/day for 13 days, orally). The DEXA group received 0.5% CMC orally for 13 days, with DEX from day 8 to 13. The AMB5 + DEX group received AMB (5 mg/kg/day, orally, 13 days) and DEXA injections from day 8 to 13. Similarly, the AMB10 + DEXA group was administered AMB (10 mg/kg/day, orally, 13 days) alongside DEXA injections. The doses and treatment period for AMB and DEXA were established based on those used in [16,66] respectively, and data from the pilot experiment were used to reproduce the effects and support the relevance of the respective treatment.

On day 14, rats were euthanized by i.p. administration of secobarbital (50 mg/kg) [66]. Blood samples were then obtained via cardiac puncture, followed by centrifugation at 3000 rpm for 10 min to isolate the serum for biochemical analysis. Animals were humanely sacrificed under anesthesia. Liver tissues were excised, rinsed in cold phosphate-buffered saline, and dried. A portion of the left lobe was fixed in 10% neutral buffered formalin for histological and immunohistochemical (IHC) examination, while another segment was preserved in glutaraldehyde for transmission electron microscopy (TEM). The remaining tissue was stored at −80 °C for subsequent homogenization in 10% phosphate buffer, and the supernatant was collected for ELISA and PCR analyses.

### 4.4. Assay of Liver-Function Parameters

Serum concentration of alanine aminotransferase (ALT) and aspartate aminotransferase (AST) were calculated via Spinreact kits (Santa Coloma, Spain) (Cat. Nos. SP41274 and MD41264, respectively) following the company’s directions.

### 4.5. Histopathological Examination

Liver tissue samples were fixed in 10% neutral buffered formalin for 24 h, then processed, embedded in paraffin, and sectioned into 5 µm slices using a microtome. The sections were stained with Hematoxylin and Eosin (H&E) for histopathological evaluation. Prepared slides were examined under a light microscope (Nikon Eclipse E200, Tokyo, Japan) to assess hepatic steatosis, inflammation, and fibrosis. Steatosis was semi-quantitatively scored on a scale of 0–3, lobular inflammation on a scale of 0–3, and hepatocyte ballooning on a scale of 0–2 according to the NAS (NASH Clinical Research Network) scoring system. Regarding scoring, the number of samples was raised to 8, as each slide contained two different sections from the same animal liver.

### 4.6. Transmission Electron Microscopy (TEM)

Liver specimens (~1 mm^3^) were fixed in 2.5% glutaraldehyde (0.1 M phosphate buffer, pH 7.4) at 4 °C for 24 h, rinsed, and post-fixed in 1% osmium tetroxide for 2 h. After dehydration through graded ethanol (30–100%), samples were embedded in epoxy resin. Ultrathin sections (~70 nm) were cut, mounted on copper grids, stained with uranyl acetate and lead citrate, and examined by transmission electron microscopy at 80 kV.

### 4.7. Assessment of Iron Homeostasis

Serum iron was assessed via the Ferrozine method using an AdipGen^®^ Life Sciences kit (San Diego, CA, USA, cat no. JAI-CFE-005). Hepatic ferritin was assessed via an Eagle Biosciences ELISA kit (Nashua, NH, USA, cat no. FRR31-K01). Hepatic transferrin and hepcidin levels were calculated via Assay Genie ELISA kit (Dublin, Ireland, cat Nos. RTDL01062 and RTFI00856, respectively). Hepatic gene expression of ferritin heavy chain (FTH1) was assessed by PCR, as revealed in Section 4.15.

### 4.8. Assessment of Oxidative Stress

Oxidative stress was evaluated by measuring hepatic levels of nuclear factor erythroid 2-related factor 2 (Nrf2) using a BT LAB ELISA kit (Shanghai, China, Cat no. E1083Ra), and 4-hydroxynonenal (4-HNE) was evaluated using ELISA kits that were obtained from Assay Genie (Dublin, Ireland, cat no. RTF101293). Hepatic malondialdehyde (MDA) levels were quantified using ELISA kits purchased from MyBioSource (San Diego, CA, USA, cat no. MBS268427) that measure MDA according to its reaction with thiobarbituric acid to form a colorimetric product detected at 532 nm. Hepatic 4-hydroxynonenal (4-HNE) levels were quantified using ELISA kits purchased from AssayGennie (Dublin, Ireland, cat no. RTFI01293). Hepatic reduced glutathione (GSH) levels were determined utilizing ELISA kits from CUSABIO (Houston, TX 77054, USA, cat no. CSB-E12144r). Hepatic glutathione peroxidase (GPX4) levels were assessed via ELISA kits from CUSABIO (Houston, TX 77054, USA, cat no. CSB-EL009869RA), and NF-κB was measured using a Cusabio ELISA kit (Houston, TX 77054, USA, cat no. CSB-E13148r). All assays were performed according to the manufacturer’s instructions, with results normalized to total protein content (mg). Each sample was analyzed in duplicate, and intra- and inter-assay variations were within the manufacturer’s recommended ranges.

### 4.9. Assessment of Lipid Stress

Serum concentrations of total cholesterol (TC), low-density lipoprotein cholesterol (LDL–C), and triglycerides (TGs) were assessed via Spinreact kits (Santa Coloma, Girona, Spain, Cat. Nos. SP41021, 41023, and MX41031, correspondingly). Very low-density lipoprotein cholesterol (VLDL–C) serum levels were calculated using a mathematical formula by dividing serum TGs/5.

### 4.10. Assessment of Ferritinophagy

Hepatic expression levels of Nuclear Receptor Coactivator 4 (NCOA-4), as a ferritinophagy biomarker, were assessed via RT-PCR technique.

### 4.11. Assessment of Autophagy

Serum calcium was assayed via Spinreact kit (Santa Coloma, Spain, cat no. MD1001065). Hepatic gene expression of calcineurin-A was assayed by PCR method. hepatic Beclin-1 levels were analyzed via Cusabio ELISA kit (Houston, TX 77054, USA, cat no. CSB-EL002658RA). Hepatic levels of autophagy-related protein light chain 3 (LC3)-II and sequestosome 1 (SQSTM1/p62) were assessed using ELISA kits (AFG bioscience, Arlington Heights, IL 60004, USA, cat no. EK721185 and Sunlong biotech, Hangzhou City, China, cat no. SL1363Ra, respectively).

### 4.12. Assay of Inflammation and Fibrosis Biomarkers

Interleukin-6 (IL-6) was assessed via Quantikine ELISA kit (Minneapolis, MN 55413, USA, cat no. R6000B), and hepatic levels of TGF-β1, collagen 1, and alpha smooth muscle actin (α-SMA) were determined using PicoKine C3RA ELISA kits (Pleasanton, CA 94566, USA, cat no. EK0514; AssayGennie, Dublin, Ireland, cat no. RTFI00677; and Cusabio, Houston, TX 77054, USA, cat no. CSB E14027r, respectively.

### 4.13. Assessment of Hepatic Endothelial and Vascular Function

Hepatic levels of prostacyclin (PGI_2_), cyclic guanosine monophosphate (cGMP), endothelin-1 (ET-1), and inducible nitric oxide synthase (iNOS) were measured as indicators of endothelial and vascular function. The concentrations of PGI_2_, cGMP, and ET-1 in the supernatants were quantified using commercially available ELISA kits (PGI_2_, Cayman Chemical, Ann Arbor, MI, USA, Cat. No. 501020; cGMP, Abcam, Cambridge, UK, Cat. No. ab133052; ET-1, Thermo Fisher Scientific, Waltham, MA, USA, Cat. No. EH1ET1). iNOS activity was analyzed via IHC as shown in Section 4.14.

### 4.14. Assay of Hepatic IL-6, iNOS, and TGF-β1 Levels by Immunohistochemistry

Hepatic IL-6, iNOS, and TGF-β1 levels were assessed by immunohistochemistry. Paraffin-embedded liver sections (5 μm) were deparaffinized, rehydrated, and endogenously peroxidase-blocked with 3% hydrogen peroxide. After antigen retrieval using EDTA buffer, sections were incubated with primary antibodies (1:100) against IL-6, iNOS, and TGF-β1 for 1 h at 37 °C. Immunodetection was performed using an HRP-DAB system, followed by visualization with DAB and counterstaining with hematoxylin.

Quantification of positive cells was performed by counting stained hepatocytes in at least five randomly selected high-power fields (400×) per section. The percentage of positive cells was calculated relative to the total number of hepatocytes per field. Alternatively, images were captured and analyzed using ImageJ software version 1.54 (FIJI, National Institutes of Health, Bethesda, MD, USA) with color deconvolution to determine the area percentage of positive staining for each marker.

### 4.15. Assay of FTH1, FABP1, Calcineurin-A, and NCOA4 Gene Expression via Quantitative Real-Time PCR

Total RNA was isolated from liver tissues using the SV Total RNA Isolation System (Promega) following the manufacturer’s protocol. cDNA synthesis was performed with a SensiFAST™ cDNA Synthesis Kit (Meridian Life Science, Memphis, TN 38134, USA). Quantitative real-time PCR was carried out using a SensiFAST SYBR^®^ No-ROX Kit (Meridian Life Science, Memphis, TN 38134, USA) on an Applied Biosystems StepOne system. Expression levels of FTH1, calcineurin-A, and NCOA-4 mRNAs were normalized to GAPDH. Primer sequences are listed in Table 1.

PCR amplification was carried out starting with an initial denaturation at 95 °C for 10 min, followed by 40 cycles consisting of denaturation at 95 °C for 15 s and annealing at 60 °C for 1 min and a final dissociation stage to confirm product specificity. All reactions were run in triplicate, and relative mRNA expression levels were calculated using the ^ΔΔ^Ct method.

### 4.16. Statistical Analysis

Quantitative data are presented as mean ± standard error of the mean (SEM). Statistical analyses were conducted using GraphPad Prism 9.0 (GraphPad Software Inc., San Diego, CA, USA). Parametric comparisons between study groups were performed using one-way analysis of variance (ANOVA) followed by Tukey’s post hoc test. Non-parametric results were compared using Kruskal–Wallis test, followed by Dunn’s multiple-comparison test. A *p*-value < 0.05 was considered statistically significant.

## 5. Conclusions

**Figure 13:**
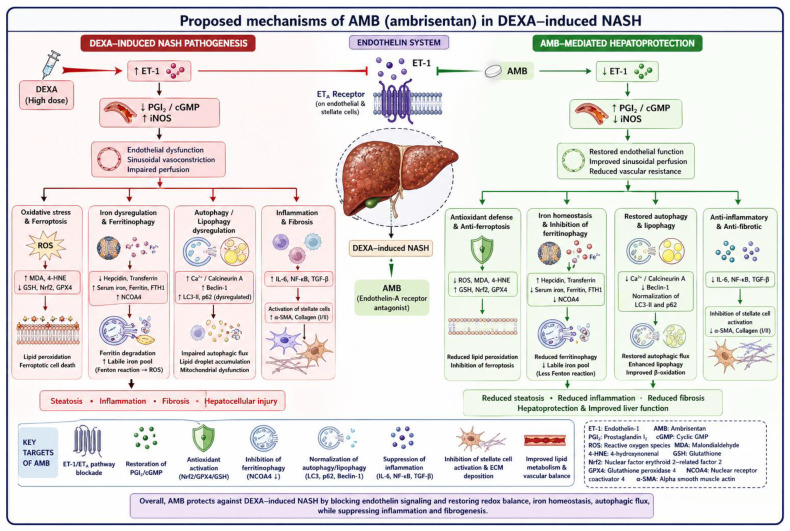
Proposed mechanisms underlying the protective effects of AMB against DEXA-induced NASH.

The evaluation of oxidative, iron, lipid, inflammatory, and vascular biomarkers underscores the multifactorial nature of DEXA-induced NASH. AMB improved DEXA-induced NASH primarily through its action on endothelin pathways, with associated reductions in inflammation and the downstream processes of ferroptosis, ferritinophagy, lipophagy, and autophagy (Figure 13).

## Figures and Tables

**Table 1:** Forward and reverse primers for PCR.

Primer	Forward	Reverse
FTH1	ATCAACCGCCAGATCAACCT	TCTCCCAGTCATCACGGTCA
NCOA-4	AGTGTCTGGGTCGGTCCAA	GTGAATCTGAGCTTTCACCTCTCGT
Calcineurin-A	AGATGGATTTGACGGAGCCAC	GCTGCTATTACTGCCGTTGC
GAPDH	TCTTCTTGTGCAGTGCCAGC	TGCCGTTGAACTTGCCGTGG

## Data Availability

The data presented in this study are available on request from the corresponding author. (please specify the reason for restriction, e.g., the data are not publicly available due to privacy or ethical restrictions).

## List of Abbreviations

**Abbreviation**	**Full Name**
4-HNE	4-Hydroxynonenal
ALT	Alanine Aminotransferase
AMB	Ambrisentan
AST	Aspartate Aminotransferase
Beclin-1	Autophagy-related protein Beclin-1
cGMP	Cyclic Guanosine Monophosphate
DEXA	Dexamethasone
ET-1	Endothelin-1
FTH1	Ferritin Heavy Chain 1
GPX4	Glutathione Peroxidase-4
GSH	Glutathione
H&E	Hematoxylin and Eosin (histological stain)
HSCs	Hepatic Stellate Cells
IL-6	Interleukin-6
iNOS	Inducible Nitric Oxide Synthase
LC3-II	Microtubule-associated protein 1A/1B-light chain 3 (LC3-II form)
LDL-C	Low-Density Lipoprotein Cholesterol
LSECs	Liver Sinusoidal Endothelial Cells
MDA	Malondialdehyde
NAFLD	Non-Alcoholic Fatty Liver Disease
NASH	Non-Alcoholic Steatohepatitis
NCOA4	Nuclear Receptor Coactivator-4
NF-κB	Nuclear Factor Kappa B
Nrf2	Nuclear Factor Erythroid 2–Related Factor 2
p62	Sequestosome-1 (autophagy adaptor protein)
PGI2	Prostacyclin
rER	Rough Endoplasmic Reticulum
T2DM	Type 2 Diabetes Mellitus
TC	Total Cholesterol
TEM	Transmission Electron Microscopy
TG	Triglyceride
TGF-β1	Transforming Growth Factor Beta 1
VLDL-C	Very Low-Density Lipoprotein Cholesterol
α-SMA	Alpha Smooth Muscle Actin
